# Aging in Flood-Prone Coastal Areas: Discerning the Health and Well-Being Risk for Older Residents

**DOI:** 10.3390/ijerph15122900

**Published:** 2018-12-18

**Authors:** Anamaria Bukvic, Julia Gohlke, Aishwarya Borate, Jessica Suggs

**Affiliations:** 1 Department of Geography, Virginia Tech, Blacksburg, VA 24061, USA; 2 Department of Population Health Sciences, Virginia Tech, Blacksburg, VA 24061, USA; jgohlke@vt.edu; 3 Urban Affairs and Planning, Virginia Tec h, Blacksburg, VA 24061, USA; baish94@vt.edu; 4 Department of Statistics, Virginia Tech, Blacksburg, VA 24061, USA; jesss93@vt.edu

**Keywords:** aging, adaptation, climate change, coastal, vulnerability

## Abstract

Coastal communities are increasingly exposed to more intense and frequent hurricanes, accelerated sea-level rise, and prolonged tidal inundation, yet they are often a preferred retirement destination for older adults vulnerable to flooding and extreme weather events. The unique physical and psychosocial challenges of older population age 65 and over may affect their level of preparedness, capacity to cope with, and ability to respond and recover from a hazard event. Despite the clear vulnerabilities of older residents living in high-risk areas when compared to younger coastal populations, there is a lack of empirical research on the integrated flood risks to this population group in the coastal context. This paper provides a holistic assessment of this emerging problem along the U.S. East Coast by measuring the exposure of older population to sea level rise and storm surge in coastal counties. It further evaluates how age-related vulnerabilities differ between rural and urban settings using the case study approach and geospatial and statistical analysis the paper also conducts a review of scientific literature to identify gaps in the current understanding of health and well-being risks to aging populations in coastal communities. The results show that older populations are unevenly distributed along the U.S. East Coast with some states and counties having significantly higher percent of residents age 65 and older living along the shoreline. Many places with larger older populations have other attributes that further shape the vulnerability of this age group such as older housing stock, disabilities, and lower income and that often differ between rural and urban settings. Lastly, our study found that vast majority of research on aging in high-risk coastal locations has been conducted in relation to major disasters and almost none on the recurrent nuisance flooding that is already affecting many coastal communities.

## 1. Introduction

Coastal communities are increasingly exposed to accelerated flooding and environmental degradation that may significantly undermine health, safety, and well-being of their residents, especially those inherently vulnerable to natural hazards, such as an aging population. Coastal fringes are being subjected to more intense and frequent hurricanes and tropical storms [1,2,3,4,5], sea level rise [6,7,8,9,10], erosion, land subsidence [11,12,13], and prolonged tidal inundation [14,15,16]. These chronic and episodic events will affect coastal lifelines, such as water supply, energy infrastructure, evacuation routes, critical facilities, assets like ports and tourist landmarks, as well as ecosystem services such as oyster farms and fishing grounds [17]. Consequently, coastal jurisdictions will require collective effort and significant resources to sustain community viability and support health, safety, and livelihoods of its residents. At the same time, many coastal counties are experiencing increased population growth and almost three times higher population densities than the national average [18]. This growth has been especially observed in coastal counties such as in Florida, which is often perceived as a “retirement magnet” or preferred living destination for aging adults age 65 and over [19,20] despite its high risk of sea level rise inundation along the shorelines [21]. Many older adults living in coastal communities intentionally choose to ‘age in place’ or “remain living in the community, with some level of independence” [22], regardless of multiple physical and environmental risks in their immediate surroundings [23]. Moreover, the Baby Boomers aged 35–44 who were predominant age group in coastal counties between 1980 and 2000 have been transitioning to the 65 and older category, likely exacerbating age-related vulnerabilities in the upcoming decades [18].

The higher concentration of older populations in increasingly hazardous coastal environments may represent an emerging public health and emergency management challenge in high-risk coastal locations due to their heightened physical and psychosocial vulnerabilities to natural hazards, disasters, and weather extremes [24,25,26,27]. Moreover, this age group often has limited mobility, greater health care access needs [28], and significantly higher mortality rates during disasters [29,30,31]. Currently, the evidence indicates that this problem exists, but there is a clear lack of understanding of its character and scope, options to attenuate it, and broader impacts on local and regional public health infrastructure and future health and wellness assistance needs. The repetitive character of accelerating hazards in coastal environments will likely put an unprecedented pressure on health and well-being of older residents. The one size fits all approach to delivery and provision of health services and programs is not sufficient to maintain and improve physical and psychosocial health outcomes among older adults in coastal locations [32,33]. Coping with more frequent and prolonged flooding among older residents can be improved by developing new contextualized paradigms in public health and health care that address the specific immediate and long-term needs of an aging population, but are also adaptive and responsive to evolving risks in coastal settings. One approach that would address these two simultaneous needs includes integration of hazard mitigation and climate adaptation planning within local jurisdictions [34,35,36].

Similarly, disaster preparedness also plays a critical role in minimizing adverse health impacts among older people during and following an extreme weather event and related flooding. In a nationwide survey of disaster preparedness among older adults, Al-Rousan et al. [37] found that older people are often inadequately prepared for disasters with a majority lacking an emergency plan, adequate information on preparedness, and sufficient backup supply of water, food, or medicine. To effectively engage with disaster preparedness, older adults should be involved in disaster response planning and receive related information in a format that makes sense to them [38]. Among older adults suffering from chronic illnesses, Kang [39] found it is more beneficial to ask them what their specific preparedness needs are rather than implement generic interventions for them. The disaster warnings should be also conveyed in language accessible to the general and particularly older audience [40]. In addition to preparedness, older adults also make distinctive choices during and in the aftermath of a disaster. For example, post-Hurricane Katrina, many older adults relied on the informal relationships and social networks to respond to impacts and were not willing to accept formal support offered or available to them [41]. In addition to physical impacts, the psychological impacts of disasters are also more pronounced in older persons than in younger adults and require a special attention from practitioners and policy-makers, especially in a form of customized risk communication [42]. For example, among people displaced by Hurricane Katrina, the psychological well-being was positively correlated to their age, physical status, and functionality within the immediate family, while the broader socioeconomic determinants like income, education, and race were less impactful on psychological status [43]. Even though many older residents of New Orleans were not directly affected by Hurricane Katrina, they were highly concerned with the indirect impacts on their community such as the demographic changes due to displacement and changes in traffic patterns, customer base serving local businesses, housing prices, number of rental properties, violent crime, drug trafficking, and gang activity [44].

Despite indicated vulnerabilities, Henderson et al. [45] show that older adults can successfully cope with various day-to-day difficulties such as interruption in basic services, access to potable water, bathrooms, clothing, and housing, overcrowding in temporary accommodations, and the lack of communications and transportation. Ashida et al. [46] found that social relationships among older adults can either impede or motivate preparedness behavior, whereas their altruistic need to help others in the community would stimulate their engagement with the preparedness. As such, this population group could still productively contribute to the development of more robust and effective disaster planning strategies that would minimize disruptions to daily life and psychological impacts. For example, older residents could provide vital child-care services that are often disrupted in the aftermath of disaster events, preventing parents to resume their regular activities and engage in recovery. They could also volunteer to disseminate disaster preparedness information in schools, libraries, and similar community venues via class modules or lectures targeting different population groups (e.g., retired residents K-12 or youth groups, religious congregations, and disadvantaged residents). With the growing recognition of the importance of socio-behavioral aspects and social capital in preparedness and emergency response, there is an increasing support for development of new planning mechanisms that would facilitate cohesion, coping capacity, and self-reliance on a community level. This concept has been propagated in federal policies as a Whole Community Approach to disaster resilience since its introduction in 2011 by the Federal Emergency Management Agency (FEMA). The Whole Community tenets emphasize the importance of shared understanding of community needs and capabilities; greater integration of all community’s resources; building cohesive social capital; and increasing individual and collective preparedness [47]. To meet this national objective, older adults can play a vital role in achieving resilient societies by contributing to all stages of disaster planning cycle, individually or as a part of collective efforts to decrease localized vulnerability.

This paper responds to the need for a more robust knowledge base about emerging challenges among older populations living in increasingly-hazardous coastal environments. It uses multi-method approach, namely geospatial, statistical, and literature gap analysis to characterize the extent and the scope of this emerging problem. The assessment measures the exposure of older residents to coastal flooding on the east coast (sea level rise and storm surge) and identifies gaps in knowledge related to health impacts of coastal chronic and episodic hazard events. The paper further explores how this problem varies between rural and urban locations under the assumption that the type and extent of factors that contribute to vulnerability differ between these two distinct settings. The key differences between rural and urban areas are discussed in greater detail in Section 3.2.2 and Section 3.2.3. This research will inform public health planning in coastal jurisdictions as well as among the regional and state public service providers by discerning the challenges affecting older population living in flood-prone areas. Many existing conventional services and programs do not have mechanisms and capacities to respond to repetitive localized health crises and emergencies, especially in remote rural communities. They also generally assume stable conditions or linear progression in demand and distribution of health support services, a norm that may be challenged by the repetitive impacts of accelerated coastal flooding. Many studies looking at issues of aging and hazards are still primarily focused on major disasters like Hurricane Katrina and Sandy [32,45,48,49,50,51] under the assumption that disruption will be short-lived, reversible, and fully restorable. In reality, many coastal localities are experiencing permanent changes in hazard propagation and risk due to sea level rise, which will likely have additive and cascading impacts on the ability of older residents to maintain their optimal health and well-being.

## 2. Materials and Methods

A geospatial assessment of the distribution of older populations was performed for 14 states on the U.S. East Coast: Maine, New Hampshire, New York, Massachusetts, Connecticut, Rhode Island, New Jersey, Delaware, Maryland, Virginia, North Carolina, South Carolina, Georgia, and Florida. The coastal county scale was determined from the NOAA’s Coastal Watershed Counties 2010 delineations [52]. The counties belong to this designation if they are contiguous to the ocean, estuaries, and connected waterways and have either “a minimum of 15 percent of the county’s total land area located within a coastal watershed or a portion of or an entire county accounts for at least 15 percent of a coastal USGS 8-digit cataloging unit” [43]. The selected 14 states incorporate 271 coastal counties. First, we measured the distribution of older population (defined as individuals age 65 and older, in percentage) in the selected coastal counties using 2015 American Community Survey 5 year estimates from the U.S. Census Bureau [53]. In the next step, we calculated what percent of this older population is exposed to coastal flooding, specifically sea level rise and storm surge. Sea level rise estimates were obtained from the NOAA’s Sea Level Rise data base [54] showing 0–6 feet inundation at the Mean Higher High Water (MHHW). The Digital Elevation Model (3 m LIDAR DEM) used to determine SLR is referenced based on the orthometric North American Vertical Datum of 1988 (NAVD88). The storm surge estimates were obtained from the Sea, Lake and Overland Surges from Hurricanes (SLOSH) storm surge data from the National Hurricane Center [55]. SLOSH models also use NAVD88 and provide estimates on storm surge heights from the historical, hypothetical and predicted hurricanes by taking into account the atmospheric pressure, size, forward speed, and tract data to model the wind field that would force the water over the land [56].

The percentage of the older coastal-based population impacted by SLR and storm surge was determined by combining a raster file of population age 65 and over for each state [57] and the sea level and storm surge shapefiles. The raster grid was used to transform irregularly shaped census block and block group boundaries into more equally distributed units. Raster data on 30 × 30 m grade allows for more accurate representation of population concentration compared to the shapefiles which assume equal distribution across the area of analysis. The sea level rise raster file was converted to a polygon shapefile to show continuous propagation of SLR since depth of inundation was not as critical as total surface area impacted. To determine the percent of older population impacted by flooding on a county level, the inundation shapefiles were combined with raster files using the zonal statistics GIS table tool in ArcGIS which identified the number of people present within the sea level rise shapefile. This was repeated for each level of sea level rise (1–6 feet) and each hurricane category (1 through 5). The data for category 5 storm surge were not available north of the North Carolina/Virginia state line, and therefore, the data on the elderly population impacted by category 5 storm surge for Cape May, NJ and Mathews, VA are not listed.

Next, we selected 10 counties among the total of 271 with the highest percentage of older residents exposed to 6 feet of sea level rise (SLR). Even though 6 feet of SLR may not affect study locations before the end of this century, we used this level of permanent inundation considering it is often proportional to the Hurricane category 1–3 storm surge in many locations. In other words, exposure of the same percentage of the older population could be imminent in the case of a hurricane event and similar to what would happen later in the future due to sea level rise. We also calculated population density and the percent of rurality from 2010 Census data. This latter dataset lists all U.S. Counties based on percent of population living in rural and urban areas with counties having less than 50 percent of the population living in rural areas being classified as mostly urban; 50–99.9 percent living in rural areas being classified as mostly rural; and 100 percent rural being categorized as completely rural [58]. Among 10 counties with the highest exposure to SLR, we selected four counties for the case study analysis based on the following criteria: counties should represent different states, and levels of urbanization with two rural (Pamlico, North Carolina, and Mathews, Virginia) and two urban (Cape May, New Jersey, and Brevard, Florida) examples. The only exception was Brevard County in Florida where only 10% of the older population would be affected by 6 feet of SLR. However, this county represents an important case considering it is located in the district well known as a preferred retirement destination in a physically highly vulnerable part of the State of Florida, where many retirees tend to reside along the oceanfront in a direct line of storm surge.

Even though a number of studies are discussing the projected population growth in the context of future climate change [59,60,61,62,63] considering the geographic scale of this study we did not account for the future population changes and rather based our estimates on the recent Census data. Using scenario-dependent assumptions, Neumann et al. [59] suggest that the total population growth in North America will be 188% in the low elevation coastal zone (LECZ) and 200% in the 100-year flood plain in the period 2000–2060. As example in Europe, Forzieri et al. [62] state that the future growth projections will contribute to only 10% increase in population and will have modest impacts on the multi-hazard risk, except for specific hazards on the local level where this effect may be more pronounced. These estimates may further differ for older populations. Among the coastal shoreline counties, the percent of population 65 years and older increased 89% from 1970 to 2010 [18]. Ortman et al. [63] suggest that the United States will continue to experience growth of its older population between 2012 and 2050 but that number may vary among young-old (age 65–74), old (75–84) and oldest-old (age 85 and over) and change over time until 2050 with the number of young-old and old-old increasing by 2030 and then leveling off. The Federal Interagency Forum on Aging Related Statistics [64] estimates this increase will be seven percent with increase of percent of population 65 years and older from 15.1% in 2015 up to 22.5% in 2050.

In the selected case study locations, we evaluated additional socioeconomic variables to determine if there are other compounding factors that would affect the vulnerability of older adults to flood exposure. For this analysis, we relied on the data from 2015 American Community Survey (ACS) 5 year estimates from the U.S. Census Bureau, including census tract data for three different disabilities (ambulatory, self-care, and independent care) and the block group data for households with individuals 65 and over, homes by year built, and household income [53]. The disability data were only available on a census tract level and were used at different scale than the data in socioeconomic descriptive analysis. The census tracts represent small contiguous county’s geographic area delineated for the presentation of statistical data [65]. The raster shapefiles for the socioeconomic variables were obtained from the Socioeconomic Data and Applications Center (SEDAC) as GEOTIFFs with shapefiles (field names A65TO79 and AOV80) included for case study states. The U.S. Census defines ambulatory difficulty as a disability in which affected individuals have serious difficulty walking or climbing the stairs; self-care difficulty in which individuals have difficulty bathing and dressing, and independent living difficulty in which individuals have difficulty doing errands alone such as visiting a health facility or stores due to physical, mental, or emotional problems. We further overlaid the layer with disabilities with the Nursing Home/Assisted Living Facilities records obtained from the Homeland Infrastructure Foundation Level Data (HIFLD, [66]). The Block Group, Tract, County, and State Boundaries were used from 2010 TIGER/Line Files from US Census Bureau (TIGER/Line Shapefiles, [67]).

In addition, we conducted correlation and regression analysis (CRA) to determine whether there is any difference between the older populations in urban and rural areas based on their socioeconomic characteristics that may affect their ability to cope with flooding. The data for this analysis was also obtained from 2015 ACS Survey for the coastal counties in 14 East Coast states [53]. To account for rurality, we used the same data set as above [58], which consists of 13,257 observations, out of which 12,984 are urban and 273 are rural. Using the logistic function in STATA, a binary regression model (logit) was used, since the response variable, urban or rural, is a dichotomous variable. The independent parameters used in the model are percent of population above 65; household income; education; ownership and occupancy rate; age of the structure; poverty rate; insurance, telephone and vehicle availability; and disability.

Lastly, we conducted a gap analysis of scientific articles to determine the scope and type of empirical research looking at the health impacts of coastal hazards on older adults. Literature was identified by searching for peer-reviewed journal articles using PubMed and EBSCOhost databases in Mar–Sept, 2017. Keyword searches were employed to find relevant studies using the following combinations of terms: (elderly OR aging OR older) AND (disaster OR flooding OR coastal) AND (health OR morbidity OR risk). Studies were further considered for inclusion if the focus included the health of the elderly and flooding or another hazard that could result in flooding in coastal settings. The content of selected articles was evaluated to determine the geographic location of the study, investigated health outcome and coastal hazard/s, methods and type of data used (primary or secondary), and key results. Findings were summarized in a tabular form.

## 3. Results and Discussion

### 3.1. Risk of Coastal Hazards for Older Population along the East Coast

**Figure 1:**
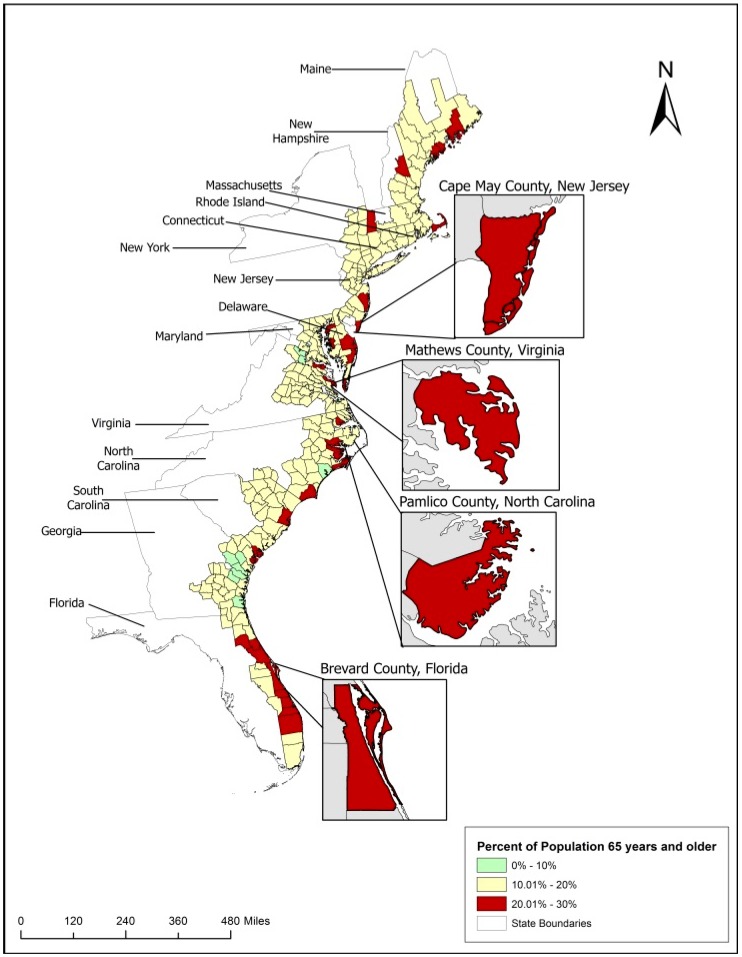
Older populations in coastal counties along the East Coast and the location of selected case studies (percent of people age 65 and older).

The percentage of the population 65 years and over ranged from 5% to over 30% in 271 coastal counties along the East Coast (Figure 1). The data for the 271 counties is normally distributed (skewness value of 1.12). Across the 271 counties an average of the population age 65 years and over is 15.4%. Delaware, Florida, Maine, New Jersey, North Carolina, South Carolina, and Virginia all have more than 20% of the coastal counties that directly border the Atlantic Ocean and/or its waterways with elderly populations making up more than 20% of the population. The county with the lowest percent of older populations is Liberty County, Georgia with 6.3% of the population age 65 and over. Lancaster county, Virginia has the highest percent of the population 65 years and over with 31.2%. Among four selected case study states, Virginia has 10.2% of counties with more than 20% of population age 65 and over, North Carolina 13.2%, New Jersey 10.5%, and Florida 36.8% respectively. Among the coastal counties in Virginia, 45.8% are adjacent to the Atlantic Ocean and/or its waterways with 22.2% of them having more than 20% of the population 65 years and older. In North Carolina, 55.3% of the coastal counties directly border the Atlantic Ocean and/or its waterways and 23.8% of them have more than 20% of the population age 65 and over. In New Jersey, 47.5% of the coastal counties are located directly on the water and 22.2% of them have 20% or more of the population 65 years and over. For Florida, only counties that are bordering or are within the coastal water basin of the Atlantic Ocean were included in the study (28% of total counties). Among them, 57.9% are located directly on the Atlantic Ocean and/or its waterways and about half have more than 20% of the population age 65 and older.

In New Jersey, Cape May has the highest percent of the population 65 years and over at 22%. Pamlico, North Carolina has the second highest in the state at 22%, behind Brunswick with 25.64%. Mathews, Virginia has the third highest in Virginia at 26%. Brevard County, Florida with 20% of the population 65 years and over is close to the average (19.1%) for the coastal counties of Florida. Florida has the highest number of coastal counties that have 20% or more of the county population age 65 and older. The geospatial analysis of current distribution patterns of older residents in coastal counties suggests that higher concentration of older population is present only in certain coastal locations that are either perceived as desirable retirement destinations with older people moving there intentionally or resulted from previous demographic trends in those areas. Such clustering of older population in areas of high exposure to coastal flooding requires customized approach to risk assessment that will account for the special preparedness, emergency response, and recovery needs of this population.

For the further case study analysis, we considered 10 counties with the population of older residents exceeding 20% and the highest risk of exposure to 6-foot sea level rise (Table 1) and added additional attributes important for the selection of case study locations such as population density, the percent of rurality based on the U.S. Census rurality classification [68], and the age of housing infrastructure. Seven out of 10 counties were mostly urban, and 3 completely rural. The older residents of Beaufort County in South Carolina have the highest exposure to 6-foot SLR (50%), followed by Cape May in NJ (35%), Pamlico in NC (35%), and Mathews in VA (30%). Among the remaining counties, less than 15% of older population will be exposed to 6 feet of SLR. We were also interested in the average age of homes in the same counties considering the age of home construction represents an important predictor of its quality and consequent ability to endure flood damage [69,70].

The susceptibility of older homes to hazard damage mostly reflects the lack of compliance with the contemporary building codes which increasingly require implementation of structural features for property damage reduction and hazard prevention. Stewart [71] indicates that the damage in the U.S. from Hurricanes Andrew (1989) and Hugo (1992) is defined by the construction vulnerability associated with the pre-1980 and post-1980 eras. Also, the homeowners of older homes with mortgages paid off are not required to purchase the flood insurance through the National Flood Insurance Program (NFIP) that is mandatory for all federally insured loans [72].

Therefore, many older homes are uninsured and may lack retrofits that would reduce flood losses from the storm events. In addition, they are often built on a slab foundation, using the dated construction methods that make the elevation and retrofits more costly and cumbersome due to issues with structural integrity, unstable foundation, and rotten joists. Beaufort and Cape May counties are both mostly urban but have a housing stock that dates from different periods: in Beaufort 27% of homes were built before 1980, while in Cape May 60% of homes were built before the same year. Pamlico and Mathews are completely rural with 47% of Pamlico homes built before 1980s and 64% in Mathews. Moreover, Mathews has 47% of homes built before 1970s and 26% before 1950, suggesting an older well established community. Northampton County in Virginia which is also completely rural has 57% of homes built before 1970 and 34% before 1950.

Other counties with higher potential impacts of SLR on older population include Worcester in Maryland, Ocean in New Jersey, Brevard in Florida, James City in Virginia, and Sussex in Delaware. Ocean and Brevard have older housing stock with 56% and 40% of housing stock built before 1980. James City and Sussex experienced a more recent development with 76% and 63% of housing stock built after 1980. Based on the number of criteria (population density, older population, rurality, age of structures, SLR exposure, and geographic location) we selected two rural counties (Cape May and Brevard) and two urban counties (Pamlico and Mathews) for case study analysis. We did not include Beaufort County, SC, considering its unique historic status as a military establishment that may not be representative of more common rural localities. Brevard County in Florida was selected regardless modest SLR inundation that would affect only 10% of older population with 6 feet increase as the exemplar of locations that serve as a preferred retirement destination and are at high risk to storm surge from tropical storms and hurricanes that would easily exceed SLR inundation projections.

We do expect that the composition and size of older populations will change in the upcoming decades. However, it is not fully clear to what extent and how this increase will differ between coastal locations and over time. Aforementioned discussion in Materials and Methods section noted the uncertainties related to estimates of future population growth and composition as well as limitations in our knowledge to account for socio-behavioral, technological, and similar societal changes that will influence adaptive capacity of older adults. Some methods, such as scenario planning offer a possibility for analyses that actually match future climate conditions with future socio-economic conditions, thus avoiding the misleading assumption that the climate of the future will meet the societies of today. Similarly, Lutz et al. [73] propose a demographic metabolism concept accounting for the multi-dimensional population attributes as effective methodological approach to forecast important aspects of societal changes that would affect adaptive capacity to climate change.

Therefore, even though the number of people in a flood zone may increase, they may have different risk perceptions and coping capacities than current residents. The dynamic nature of population and demographic changes can be observed in many different settings and under various stressors that drive the change, e.g., environmental degradation, disasters, and economic difficulties. For example, such trends have been observed in the Miami metropolitan area with the older people moving out of the area further inland and young professionals moving into the region [74]. Considering there is insufficient evidence on how age-related population increases would be distributed across the coastal counties and vary over longer time intervals, we rely on the present numbers in analysis. At the same time, we are cognizant that more research on population dynamic methodologies and granular population projections would be helpful to better understand the impacts of population growth and demographic changes on risk propagation in coastal locations.

### 3.2. Case Studies

#### 3.2.1. Exposure

Currently, the planning for hazards and disasters in the U.S. is still mostly focused on disaster preparedness and hazard mitigation in relation to episodic acute hazard events with less attention dedicated to long-term and persistent impacts such as sea level rise and nuisance flooding. Table 2 shows the percent of the older population potentially exposed to different levels of sea level rise and storm surge of hurricane categories 1–5. For rural Mathews County, 6 ft sea level rise would affect almost 19% of the older population, with almost threefold increase in exposure from the percent impacted at 1 ft of SLR inundation. For storm surge, a Category 1 hurricane would affect the same percent of older residents as would 4 ft of SLR, while a Category 4 event would impact almost twice as many older residents than what would be affected with 6 ft of SLR. In rural Pamlico County, the impacts of SLR would be more pronounced with each additional foot of increase, growing more than nine-fold from 4.5% of impact at 1 ft to 41.7% of impact at 6 ft. The storm surge associated with the weakest Category 1 hurricane would already impact 31% of older residents, while Category 5 surge would affect the majority of coastal older residents (n = 78.7%). In urban Cape May, 1 ft SLR will affect 11.7% of population age 65 and over and progressively increase to 39.4% at 6 ft. In the same County, 36.6% of older population would be impacted by Category 1 hurricane and more than 70% by Category 3 and 4 events. Of all selected case study locations, Brevard would be the least impacted, and even under the worst case scenario of SLR and storm surge only 9.6% and 21.1% of older adults respectively would be impacted. In Mathews, Pamlico, and Cape May counties, the storms surge caused by even the weakest storm would put at risk more than half of the older residents, with the exposure being most severe in Pamlico and Cape May where even Category 3 storm would affect over 70% of older residents. This comparison highlights the importance of a multidimensional approach to the risk assessment of coastal hazards that would account for different flood-exposure scenarios and related uncertainties to ensure that communities develop a robust resilience for all eventualities.

#### 3.2.2. Rural Context

The research looking into rural disaster preparedness and response is more limited than that for urban areas, and almost non-existent for the assessment of persistent stressors such as flooding and erosion. Disaster resilience also greatly varies between urban and rural typologies and is generally higher in urban areas than in the rural locations although the underlying determinants of higher resilience significantly differ between diverse rural and urban contexts [75]. Rural areas have unique challenges that influence their planning for disaster preparedness, such as a lack of resources, access to training opportunities, the organizational capacity to manage resources, reliance on agriculture as the main economic base, and problems with timely communication between emergency responders and residents [76]. The disaster impacts and recovery process also vary between rural and urban settings, especially in post-disaster redistribution of vulnerabilities and progress with the recovery process [77]. In the rural context, people are often more self-sufficient and self-reliant on their inherent skills and capacities to deal with hazards and disasters. From the preparedness perspective, this attribute may have its advantages and disadvantages. On the positive side, rural residents can better identify their immediate needs, organize and coordinate actions, and contribute to response activities; while on the negative side, their lower socio-economic status, outflux of younger and educated residents, and lower educational attainment may inhibit preparedness [78]. They are also generally more impoverished, have more racial inequality and health disparities, limited professional training, higher aging population, and high rates of out migration [75]. Further, rural communities have more dispersed residential patterns, fewer resources, and limited access to contemporary equipment, technology, and training [79]. Rural populations may also feel more isolated, have limited family and social support, and face overall more challenging physical and social environments than their urban counterparts [80] who are more dependent on the institutional support to manage their environment. Some of the aspects of rural counties that contribute to their lower disaster resilience are influenced by their proximity to large metropolitan areas, main transportation routes, and industrial and tourist destinations [75]. To explore the exposure and vulnerability of coastal older rural populations in greater detail, we performed geospatial analysis of socioeconomic factors that contribute to their susceptibility to flooding in two case study locations, Mathews County in Virginia and Pamlico County in North Carolina.

*Mathews County, Virginia*. Mathews County covers 86 square miles of land and 166.3 square miles of water. It has a population of 8779 (2017 estimates), 84.6% homeowners, mostly white residents (87.4%), 30.8% of people age 65 and over, average households size of two persons (versus 3 in Virginia), and median household income of $64,049 (2016) [81]. It is surrounded with water on three sides; adjoining the Piankatank River to the north, the Chesapeake Bay to the east, and Mobjack Bay to the south [82]. Considering that all areas of the County are within a 2 mile distance from more than 150 miles of shoreline and its numerous inlets, bays, and creeks, the main livelihood activities include farming, fishing, oystering, and crabbing [82]. Even though the County is highly vulnerable to tidal flooding, erosion, stormwater runoff, and storm surge from hurricanes and northeasters, it is likely that many miles of pristine shoreline will experience development pressures in the near future [82]. The limited protection from floods include bulkheads, seawalls, jetties, sand dunes, and non-structural measures such as zoning codes and building ordinances [82].

*Pamlico County, North Carolina*. Pamlico County covers 337 square miles of rural area and 229.3 square miles of water. It has population of 12,689 (2017) with 75.7% homeowners, mostly white residents (77.5%), 28.9% of population 65 years and over, two people per household and median income of $43,808 [81]. It is located in the tidewater region on a peninsula adjacent to the Pamlico River on the north and Neuse River on the south with many waterways shaping the landscape of this county [83]. The Pamlico County is connected to the inland destinations via two main roadways serving as main communities arteries for majority of residents (75.3%) who commute alone to work by vehicle on average 26.3 min [84]. The long-standing culture and history of this County “with deep ties to water” and livelihood based on fishing and seafood industry was shaped by decades of hardship and survival in the face of “hurricanes, floods, disease, economic depression, and disadvantages of physical and social isolation”, which “produced a stubborn independence and sense of self-sufficiency in the residents, who traditionally have been slow to accept social or political change” [83]. The Pamlico County experienced significant flooding and erosion during Hurricane Matthew, Irene, and other events which emphasized its hazards, including wind-driven tidal surge that pushes water up the numerous rivers and creeks; beach and dune erosion; chronic flooding of structures and roadways that results in regular inaccessibility; wind damages and frequent power outages; salinization and resulting loss of timber; and sediment and debris depositions [84]. An aging and inefficient drainage system often get clogged by deposition of debris during extreme weather events and further exacerbates flooding and contributes to mosquito infestations and resulting public health issues [84].

*The Geospatial Analysis*. The analysis shows that in both rural counties, older residents tend to live in higher numbers in the census blocks directly adjacent the open water. The Mathews County has more than 50% of households with individuals 65 years and older living in the physically most exposed census blocks facing open Chesapeake Bay waters, close to the Bay’s convergence with ocean. The same blocks have lower per capita income of $20,000–40,000 and more than 50% of homes built before 1980. In Pamlico County, all five census blocks with more than 40% of households with older residents are facing the open waters and have low to medium income per capita. Out of nine census blocks adjacent to the waterfront, four have more than 50% of housing stock older than 1980, four 40–50%, and only one has less than 30%. These two examples show that not only older individuals tend to live closer to the coastline, but also in census block groups with lower per capita income and older homes. This broad profile may adversely affect their flood risk; they may have competing priorities between supporting the growing costs of their health care that tends to be higher for this age group and investing resources in structural flood proofing and retrofits to meet present-day flood sensitive building codes. The older homes tend to be grandfathered-in or exempt from meeting the newer building codes and participating in National Flood Insurance Program (NFIP), which leaves them more vulnerable to flood damages.

#### 3.2.3. Urban Context

The urban areas are especially vulnerable to the accelerating coastal flooding due to their high population densities, interdependencies between social, economic, and built systems, as well as valuable assets and critical infrastructure that are vital for the urban functionality. They are also highly developed, have aging infrastructure and buildings, and often face other urban issues such as the lack of affordable housing, strained transportation system, legacy of industrial pollution, and various socioeconomic problems. The high residential densities, aging urban forms, agglomeration of places where people amass, the interconnected infrastructure and services, and overall complexity and potential for cascading events in the cities shape the disaster resilience of urban centers [85]. Other characteristics that define urban profiles include more fragmented and diversified social fabric; economic activities in manufacturing, services, and administration; higher education; easy access to amenities, information, and training opportunities; lower sense of community, low fertility and mortality; more liberal political environment; ethnically and cultural diversity; and higher in and out migration [86]. In contrast, the rural settings are less densely populated, with less educated but more self-sufficient and socially cohesive residents that are highly dependent on the vehicles for transportation. Rural areas are also experiencing economic outmigration of affluent and younger population to places of better employment opportunities which are often located in the urban centers. To better understand the exposure and social vulnerability of older adults in urban context, we performed a geospatial analysis of socioeconomic variables in two urban case study locations—Cape May in New Jersey and Brevard in Florida.

*Cape May, New Jersey*. The Cape May County faces the Atlantic Ocean on the east and Delaware Bay on the west, covering 255 square miles of land and 365.1 square miles of water. The County population is 93,553 (2017; 83% urban, 17% rural) with significantly higher number of renters (23.6%) than in our two rural case studies, mostly white residents (85.2%), 25.6% of population age 65 and over, average household size of 2 people (versus 3 statewide), and the median household income $59,338 (versus 72,222 for the state) [81]. The culture and history of this County, with its many ecologically valuable lagoons, marshes, and wetlands, is highly shaped by the historic presence of tourism and the resort industry driving the dense and expensive development of coastline fringes, including the vacation homes, bed-and-breakfast lodging, hotels, and support facilities [87]. The workforce that supports the economic activities in tourism lives further inland, in more affordable districts [87]. Some of the key vulnerabilities and challenges facing the Cape May County include the Nor’easters which are highly damaging and more frequent than tropical systems [88] and dense oceanfront development with disproportionally higher numbers of transitory residents and tourists during the summer hurricane season with limited knowledge of local hazards [87]. The “lucrative location” of this County, already crowded with housing, manufacturing, and tourist-related assets, is likely to continue in its investment appeal and population growth [89]. This is especially concerning, considering that the City of Cape May, the main seaside resort in this County, is located in the low laying area with highly sensitive environmental features and fragile dunes, all of which would further exacerbated flooding should they be compromised to accommodate future development [90].

*Brevard, Florida*. The Brevard County is located on the east coast of Florida along the Atlantic Ocean, covering 1018 square miles of land and 538.8 of water area and including several barrier islands. The population of 589,162 (2017) is highly urban (95%) with 28.3% of mostly white residents (83.4%) living in rental accommodations [81]. The average household size is 2 individuals, the median household income is $49,914 (2016), and the population 65 years and older is 23.3% [81]. The County covers area also known as Florida Space Coast with the pristine beaches at high risk of erosion [91]. Brevard County is a home to many larger urban communities such as Palm Bay, Melbourne, Titusville, as well as tourist attractions such as John F. Kennedy Space Center and federal partners namely Cape Canaveral Air Force Station, Patrick Air Force Base, and National Aeronautics and Space Administration (NASA). The population of Brevard County and residential, commercial, and industrial development grew significantly over the last few decades, mainly along the barrier islands and the eastern shore of the Indian River [92]. The County is highly prone to flooding from heavy rainfall, especially along the rivers, streams, creeks, and channels, to storm surge resulting from hurricanes and tropical storms, and to tidal surges exacerbated by high winds [92]. The existing flood control features, namely levees and dikes, are structurally flawed and may not offer adequate protection from the rare events bring the extensive flooding [92]. For one of the counties highly vulnerable barrier island communities, the City of Satellite Beach, options are very limited; the island is consisting of porous and permeable sand that will allow flow of water beneath or behind any structural flood protection features like dikes and seawalls making them obsolete [93]. Considering its highly developed footprint and low lying topography, the retreat to higher ground is also not a realistic response [93].

*The Geospatial Analysis*. The analysis of the Cape May County shows that a number of households with older residents are concentrated in the census block clusters along the Atlantic Ocean open waters. The majority of census blocks facing water have more than 40% of households with individuals 65 years and older. The same blocks also have a higher per capita income and an older housing stock. Overall, the majority of blocks have a large proportion of housing stock dating back before 1980s (more than 40% of all homes). In Brevard County, the tracts with higher percent of households with older residents are more inter-dispersed but still have in the majority of places 30% or more of households with older residents. The older housing is clustered in the larger population centers with a significant proportion of newer construction built in between established communities, reflecting the recent rapid growth this County has experienced (from 398,978 in 1990 to 543,376 in 2010, [94]).

The comparison of case studies in rural and urban locations suggests that in both coastal contexts, older populations tend to aggregate along the waterfront. The differences between rural and urban settings are evident from their socioeconomic attributes, with rural examples having older population living in census blocks with lower income and high percent of older housing and urban cases having older adults residing in blocks with higher income and more diversified land use patterns. The analysis suggests that physical exposure to coastal flooding by proximity to hazard may be similar for rural and urban residents; however the consequences for the older population may significantly differ between them. In rural settings, the limited resources and more homogenous housing stock dating from the same era shape their adaptive capacity and disaster risk on a more extensive level. In urban places, the value of assets, higher incomes, and more dispersed patterns of housing dating to different times, may help distribute the type and extent of loss and impacts on older population. To better understand the dichotomy between coastal rural and urban settings on a broader scale, we performed additional statistical analysis for all coastal counties on the East Coast.

### 3.3. Rural versus Urban Socioeconomic Disparities in Coastal Counties

In this section, we evaluate disparities between urban and rural coastal settings that may compound the vulnerabilities to coastal flooding among older residents. Table 3 shows that urban census tracts are more likely to have higher percentages of the population above 65 years of age. Compared to urban census tracts, rural census tracts are more likely to have higher disability rates. On the contrary, the percent of the population without health insurance and without a vehicle is more likely to be higher in urban census tracts than in the rural census tracts. With respect to housing, urban census tracts are more likely to have higher occupancy rates and lower home ownership rates. It is important to note that none of the odds ratios suggest strong effects, suggesting that urban and rural coastal communities have unique characteristics not adequately captured by these sociodemographic variables. In terms of vulnerability of older residents in urban versus rural coastal communities, this analysis suggests some potential hypotheses to explore in future studies where individual- or parcel-level data is available. For example, rural residents may be less likely to evacuate due to higher likelihood of home ownership; however they may also be more likely to become injured during a flooding event due to higher rates of disability. On the other hand, lower rates of vehicle availability and health insurance coverage in urban census tracts could suggest reduced likelihood of evacuation and seeking medical care.

The results of correlation analysis (Table 4) show a moderate negative correlation between percent of older population (age 65 and over) and percent of population with less than a high school diploma, indicating that census tracts with a higher percent of older populations also have higher high school completion rates. Moderate positive correlation is observed between older population and homeownership with older residents more likely to own a home. Similarly there is a high positive correlation between percent of older population age and population with disability. The findings also suggest that urban census tracts have a higher likelihood of having more homes built before 1980. This result further strengthens the output of the logistic regression model i.e., it is consistent with the outcome that urban census tracts are more likely to have older housing.

### 3.4. Health and Safety of Older Adults Living in Flood-Prone Coastal Fringes

**Figure 2:**
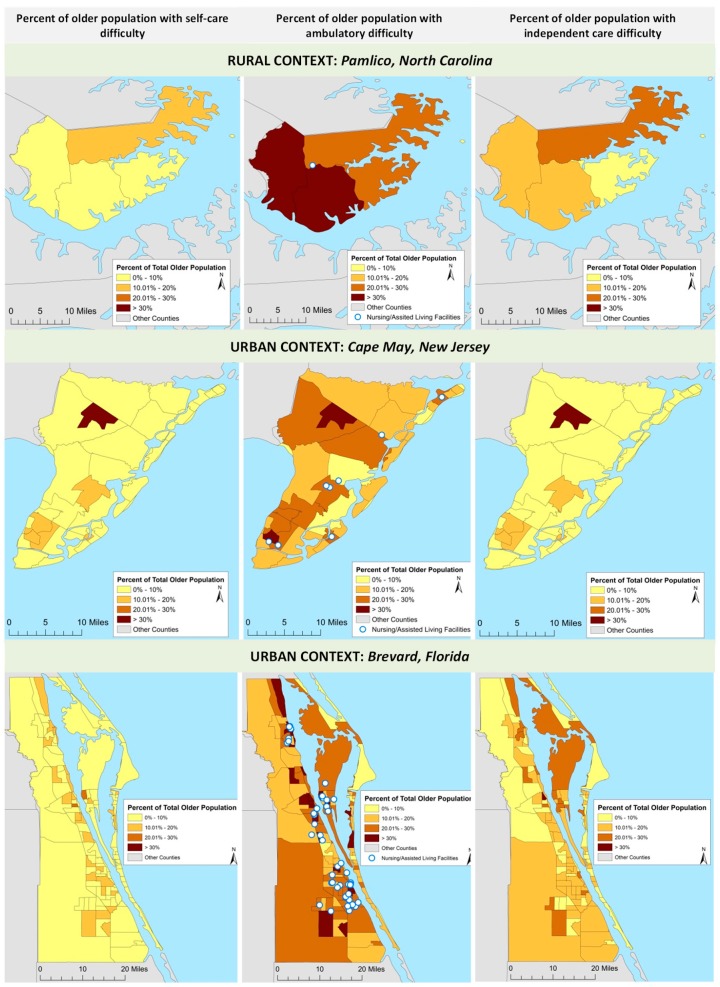
Tract level distribution of self-care, ambulatory, and independent living disabilities in three case study locations.

Being older and also having physical disabilities can present an additional challenge for individuals in this age group, further limiting their ability to engage in preparedness activities [37], evacuate, and cope with impacts in the aftermath of events. To better understand how presence of such disabilities might affect the risk of exposure to coastal flooding, we evaluated their distribution among the coastal older population (Figure 2). The disability records for Mathews County, Virginia do not have any significant variation in the incidence of three measured disabilities and were therefore not included in the mapping products. Pamlico County in North Carolina had a lower percent of older residents with self-care difficulty, and slightly higher percent of independent care difficulty. Ambulatory difficulty, in which affected individuals have a serious difficulty in walking or climbing the stairs, is the primary cause of disability within older residents in Pamlico County.

Out of four census tracts in Pamlico County, in two, more than 30% of older residents had ambulatory difficulty, and in the other 20–30%, indicating a potentially significant problem during flood events, when older residents may have to evacuate or climb to higher ground. The lack of multiple nursing and assisted living facilities in Pamlico County presents a potential issue for recovery for those with ambulatory difficulty. With a single nursing/assisted living facility in the county, limited support care options may hinder the ability of the older population to recover post disaster in comparison to the urban counties, Cape May and Brevard, which have multiple facilities to provide post-disaster assistance. In Cape May County, the majority of census tracts have lower incidence of self-care difficulty except the tract incorporating Woodbine. Brevard County has very low incidence of self-care difficulty, mostly under 10% with only 20 blocks between 10–20%, and somewhat higher rates of independent care difficulty with the majority of blocks having 10–20%, followed by 16 with 20–30%, and one with more than 30% coverage. The highest percent of the older population affected by measured disabilities is for ambulatory difficulty, with many areas adjacent to shoreline having 20–30% and 30–40% of incidence. Brevard County also has a significantly higher number of nursing and assisted living facilities located in close proximity to waterfront areas, compared to Pamlico and Cape May counties.

The spatial information outlined above on percent of population with disabilities is an important source of information for hazard mitigation and climate adaptation planning in terms of factors associated with higher risk of adverse health outcomes. However, a more thorough characterization of actual adverse health outcomes seen during and after disasters and the role of underlying predisposing conditions would significantly improve risk-reduction interventions. Table 5 summarizes current knowledge on health outcomes in elderly populations associated with coastal flooding. Lane et al. [95] present a logic model of potential health impacts of coastal storms, separating out short-term and long-term health outcomes from secondary hazards including power outages, wind, and storm surge and precipitation. Most of the current evidence is derived from analyses after major acute natural disasters including hurricanes, tsunamis, and typhoons in the North America (New Orleans & New York) and Asia (Japan, Sri Lanka, Indonesia, Bangladesh, & China).

Retrospective analysis of medical records or survey methods have been the primary data sources used to determine health impacts during and after flooding events. While overall mortality and mental health impacts in elderly populations have been fairly well characterized following flooding events, other specific impacts, including injuries, complications following reduced chronic disease management and power outages, and infectious diseases have been less well studied, therefore are more difficult to explicitly consider and incorporate into hazard mitigation and climate adaptation planning.

Previous researchers have delineated some important health considerations in planning specifically for older populations in coastal areas. Kleinosky et al. [114] emphasize the importance of collecting baseline data for older adults with disabilities and special equipment in areas that are geographically at risk for flooding or natural disasters that could lead to flooding, which can be accomplished at the census tract level through use of freely available data from the American Community Survey, as demonstrated in this paper. According to the National Council on Aging [115], nearly 92% of seniors have at least one chronic non-communicable disease (NCD), and roughly 77% have two or more. The most common NCDs include: heart disease, cancer, stroke, and diabetes—and cause almost two-thirds of all deaths each year. Diabetes, in the United States, impacts 12.2 million people over the age of 60, which is around 23% of the population. Moreover, almost 90% of elderly Americans are prone to hypertension [102].

Ryan et al. [116] provides a comprehensive review of non-communicable disease complications during and following natural disasters. Importantly, treatment management and interrupted care of NCDs during previous natural disasters that involved flooding has been shown to lead to increases in severe complications, hospitalizations and sometimes premature death. Future research and intervention strategies need to address care and management complications during extreme weather events, as NCD rates continue to rise. Fewer studies have been conducted examining infectious disease after a natural disaster that includes flooding, however diarrheal illness, specifically associated with vibriosis, was reported after Hurricane Katrina [117,118]. Additionally, an analysis of emergency department visits pre, during and post tropical cyclones and hurricanes in Florida found increases in Cryptosporidiosis, salmonellosis, and vibriosis, particularly in older adults [119] and analyses after Superstorm Sandy found similar risks [120]. Public health campaigns have been used to prevent additional food and waterborne illnesses during and following flooding, for example, during and following Hurricane Harvey [121].

## 4. Limitations

While the above analyses use information from a variety of sources, one major limitation of this research is the lack of individual- or parcel-level data. Future studies should focus on collecting and analyzing granular data from older residents in a wide range of urban and rural coastal locations to identify quantitative and qualitative attributes that define their vulnerability to coastal flooding. This research may benefit from primary data that would clarify the role of contextual factors on the individual- and household-level resilience to flooding for different subgroups of older populations. In addition, even though the comparison of two distinctly different hazards, storm surge and sea level rise, is an important indicator of a need for multipronged approach to hazard preparedness, it merely indicates one potential scenario rather than other possibilities reflecting individual coping capacities of older adults. And lastly, even though out paper focuses on older population age 65 and over, it is important to note that not all older adults are the same. Many older individuals have local knowledge and life experiences, as well as psychological resilience to deal with prolonged or repetitive hazard exposures and effectively engage in disaster preparedness and adaptation.

## 5. Conclusions

This paper addresses an emerging issue of vulnerability of older adults to accelerating coastal flooding. Coastal hazards such as tidal inundation, sea level rise, storm surge, and high winds are capable of exerting direct and indirect impacts on places where older people live and places where they need to go to meet their health and well-being needs. They can aggravate physical health of older adults by reducing their mobility and access to health services, as well as by putting them in situations where they have to operate in unsafe conditions or engage in actions that are not compatible with their physical capabilities (e.g., wade through the floodwaters, drive in the dark, or move heavy objects to minimize property damage). Hazard exposure may also affect psychosocial well-being of older adults by, for example, promoting isolation due to disruption in communications, power grid, and accessibility to public facilities. The role of well-being on one’s overall psychosocial health has been increasingly recognized in the literature as an important contributor to the overall health of older individuals. It has been associated with positive relationships with others; purpose in life; realization of potential and self-acceptance [122]; individuals’ perception of their current situation and their aspirations [123]; and lack of distress and dysfunction [124].

The exposure to recurrent flooding and consequent strain of dealing with repetitive damages, loss of belongings, changes in the demographic community profile, restricted accessibility to gathering places, and other impacts can all affect the well-being of older coastal residents. Even though some studies suggest that older residents cope better with disasters and are more resilient during the recovery process [49,125,126,127], Eisenman et al. [128] observed after Hurricane Katrina that “social isolation, even in the midst of a large community, prevents many older people from receiving warning signals or asking for help, rendering them invisible to rescue teams”. Thus, it is unclear how this protective effect would be challenged by recurring hazard events and where the threshold would be beyond which it would diminish. Similarly, less is known how life experiences and acquired skills contribute to the sense of self-sufficiency and confidence in coping capacity that may prompt many older adults to stay in place regardless of the risk.

In this study, we found that some states on the U.S. Eastern shores have a higher population of older adults living in areas prone to coastal flooding. Namely, Delaware, Florida, Maine, New Jersey, North Carolina, South Carolina, and Virginia all have more than 20% of the coastal counties that directly border the Atlantic Ocean and/or its waterways with elderly populations making up more than 20% of the population. These higher concentrations may form pockets of vulnerability: even though New Orleans, Louisiana had only 15% of older population age 60 and over prior to Hurricane Katrina, they represented over 70% of all victims with many more experiencing a health decline due to disruption in medication treatment or lack of access the medical equipment necessary for independent living [29]. The examples from earthquake, tsunami, and heat wave disasters show that the majority of disaster fatalities do occur among individuals older than 60 years with those over age 74, being at the highest risk due to frailty, cognitive impairment, and limited mobility [129]. Even though chronic nuisance flooding is less likely to lead to immediate fatalities, it will still pose persistent complex challenges for older adults.

For example, in our paper, we identified that many counties with the highest percent of older population also have an older housing stock that is more prone to damage and more expensive and difficult to retrofit. Further, we found that a number of counties affected by future sea level rise are located in rural areas where the older population is already predisposed to limited availability of health care and support services, isolation, and access to information. In two rural case study counties, we found that older residents tend to live not only closer to the waterfront, but often have a lower per capita income and older homes. In urban settings, many older adults also live along the coast and in older homes, but generally have higher income than the same population group in rural counties. Our analysis suggests that urban census tracts have a higher percent of older population, higher occupancy and renting rates, and higher population without health insurance and vehicle. On the contrary, coastal rural census tracts have lower educational attainment and higher disability rates. A more comprehensive understanding of these general predispositions among rural and urban coastal populations may help identify specific support needs that could increase the coping capacity, engagement in disaster preparedness, and overall resilience of this population group. For example, even though renting is often identified as an indicator of lower vulnerability, it may also support mobility and allow tenants to find alternative housing options in response to progressive flooding. In urban environments, the new resiliency-building programs should focus on accessibility and reliability of public transportation during hazard event and on policy changes that will allow older residents to adjust their circumstances in synergy with their evolving age-related health and well-being needs and with hazard propagation. In rural coastal settings, adaptive interventions should focus on risk communication and innovative financial mechanism such as microloans, tax deductions, or rebates that would support people’s self-sufficiency and capacity for self-organization in resilience-building efforts. In some cases, that may mean only providing technical assistance and non-monetary support such as labor, materials, or equipment.

Considering that the extent of SLR and storm surge impacts will vary greatly between different locations, the decision-making should take a two-pronged approach to adaptation planning, one that addresses the acute but less predictable episodic events and another one that uses the long-term visioning and scenario planning to support adjustments in land use, delivery of services, and sitting of critical facilities. Both rural and urban counties have high rates of ambulatory and independent living disabilities, with many more nursing homes/living assisted facilities located in urban centers. Flooding in coastal communities is often caused by a complex set of circumstances, many of which involve an unsustainable legacy of land use and development, aging infrastructure, increased risk of precipitation and flooding from the sea and unique socioeconomic dimensions. Thus planning for adequate health and well-being support of older residents should be comprehensive and explore multi-hazard scenarios that also include cascading events and secondary impacts, such as loss of power and access to health facilities.

Lastly, we found that while significant research has been conducted outlining increased short and long-term health risks following disasters, very little research has been conducted on recurrent nuisance flooding that had not resulted from a major weather event. It is unclear whether results from major disasters can be extrapolated to lower level of coastal flooding and whether preparedness strategies for major events will also lead to better outcomes following chronic flooding events. In addition, while much research has been devoted to characterizing and quantifying the health risks associated with natural disasters, effectiveness of intervention strategies are more difficult to study due to the episodic and unpredictable nature of extreme weather events. Future research in this area could take advantage of areas prone to coastal flooding to evaluate intervention strategies over shorter periods of time that may be applicable to larger flooding events associated with more rare extreme weather events.

## Figures and Tables

**Table 1:** Ten counties with the older population over 20 percent and the highest exposure to 6-foot sea level rise, including the percent of rurality and the age of homes.

County	State	2010 Census Total Population	Population Density	Percent Rural	Age 65 and over	Homes Built since 1980	Homes Built before 1980	Sea Level Rise
**Beaufort**	SC	162,233	281.5	19.6	20%	73%	27%	50%
**Cape May**	NJ	97,265	386.9	17.5	22%	39%	60%	35%
**Pamlico**	NC	13,144	39.1	100	22%	53%	47%	35%
**Mathews**	VA	8978	104.5	100	26%	36%	64%	30%
**Worcester**	MD	51,454	109.9	35.5	23%	55%	45%	15%
**Northampton**	VA	12,389	58.5	100	22%	28%	71%	15%
**Ocean**	NJ	576,567	917.0	2.9	21%	43%	56%	10%
**Brevard**	FL	543,376	535.0	5.1	20%	61%	40%	10%
**James City**	VA	67,009	470.4	15.9	21%	76%	24%	10%
**Sussex**	DE	197,145	210.6	41.3	21%	63%	37%	10%

**Table 2:** Percent of population 65 and over impacted by sea level rise or storm surge in case study locations.

Case Studies			Percent of Impacted Population Age 65 and Over										
			Sea Level Rise						Hurricane Storm Surge				
County	State	Profile	1 ft	2 ft	3 ft	4 ft	5 ft	6 ft	Category 1	Category 2	Category 3	Category 4	Category 5
Mathews	VA	Rural	5.5%	8.4%	10.7%	11.3%	15.8%	18.9%	11.7%	24.3%	31.4%	34.7%	N/A
Pamlico	NC	Rural	4.5%	16.5%	23.9%	26.2%	35.3%	41.7%	31.0%	63.4%	74.8%	77.5%	78.7%
Cape May	NJ	Urban	11.7%	15.1%	17.7%	25.3%	32.2%	39.4%	36.6%	52.5%	73.0%	76.9%	N/A
Brevard	FL	Urban	3.6%	4.0%	4.6%	5.4%	7.3%	9.6%	1.4%	3.4%	7.8%	14.6%	21.1%

**Table 3:** Regression analysis of census tracts in coastal counties based on the level of rurality.

Variables	Odds Ratio	Percent	p > z	95% Conf. Interval	
% Population above 65 years of age	1.07	6.58	**0.00**	1.04	1.09
Household income	1.00	0.00	**0.00**	1.00	1.00
% Less than high School	0.99	−0.21	0.85	0.98	1.02
% Homeowners	0.96	−4.25	**0.00**	0.95	0.97
% Occupancy	1.07	6.94	**0.00**	1.06	1.08
Median year structure built	1.00	−0.18	**0.00**	0.99	0.99
% Living in poverty for age 65 and over	1.00	0.49	0.63	0.98	1.02
% No health insurance coverage for age 65 and over	1.25	24.71	**0.00**	1.11	1.40
% No telephone service for age 65 and over	1.13	12.91	**0.16**	0.95	1.34
% Age 65 and over with a disability	0.93	−7.42	**0.02**	0.87	0.99
% Occupied housing with no vehicle available	1.04	4.44	**0.00**	1.02	1.07

Note: The values in bold have a strong positive or negative correlation.

**Table 4:** Correlation analysis of variables in census tracts in coastal counties.

		1	2	3	4	5	6	7	8	9	10	11
**Density**	1	1.00										
**Population 65 and over**	2	−0.19 0.00										
**Less than high school**	3	0.36 0.00	−0.23 0.00									
**Household income**	4	−0.16 0.00	−0.02 0.03	−0.59 0.00								
**Owner occupied**	5	−0.55 0.00	0.35 0.00	−0.54 0.00	0.54 0.00							
**Renter occupied**	6	0.55 0.00	−0.35 0.00	0.54 0.00	−0.54 0.00	−0.99 0.00						
**Property age**	8	0.00 0.60	−0.01 0.45	−0.02 0.05	0.01 0.17	−0.01 0.13	−0.02 0.00					
**Poverty**	9	0.40 0.00	−0.15 0.00	0.58 0.00	−0.48 0.00	−0.59 0.00	0.58 0.00	−0.01 0.18				
**No insurance**	10	0.10 0.00	−0.15 0.00	0.20 0.00	−0.09 0.00	−0.19 0.00	0.18 0.00	0.02 0.07	0.17 0.00			
**No telephone**	11	0.03 0.00	0.16 0.00	0.18 0.00	−0.21 0.00	−0.12 0.00	0.12 0.00	−0.02 0.01	0.21 0.00	0.02 0.04		
**Disability**	12	−0.12 0.00	0.83 0.00	−0.02 0.00	−0.23 0.00	0.16 0.00	−0.16 0.00	−0.02 0.05	−0.04 0.00	−0.13 0.00	0.21 0.00	
**No vehicle**	13	0.80 0.00	−0.19 0.00	0.51 0.00	−0.33 0.00	−0.73 0.00	0.74 0.00	−0.00 0.81	0.58 0.00	0.10 0.00	0.12 0.00	−0.04 0.00

**Table 5:** Health outcomes associated with coastal flooding in older populations.

Health Outcome	Data Sources Used	Location/Source of Flooding	Main Results	References
**Physical Health**				
Mortality	Medical records, social security records, survey instruments	Gulf Coast-hurricanes, Japan-tsunami, Sri Lanka-tsunami, Philippines-typhoon, Indonesia-tsunami	↑ mortality rate in elderly women, evacuation messages may not be received by elderly, evacuation hazards should be weighed against shelter in place,	Nomura et al. 2016 [96]; Davies & Hemmeter 2010 [97]; Ching et al. 2015 [40]; Rofi et al. 2006 [98]; Jonkman et al. 2009 [99]
Non-fatal injuries	Health registry from cohort study	New York-Hurricane Sandy	↑ incidence during evacuation and clean-up	Brackbill et al. 2014 [100]
Pneumonia	Medical records	Japan-tsunami	↑ incidence, hospitalizations, and mortality. ‘Tsunami lung’ likely via water aspiration	Shibata et al. 2016 [101]
Influenza	Medical records	Japan-tsunami	↑ incidence in elderly in evacuation centers	Kamigaki et al. 2014 [102]
Chronic disease management	Interviews	New Orleans-Hurricane Katrina	↑ missed dialysis, ↑risk of hospitalization	Anderson et al. 2009 [103]
Myocardial infarction	Hospital records	New Orleans-Hurricane Katrina	↑ 3-fold incidence 2 years after hurricane	Gautam et al. 2009 [104]
power outage related deaths	Medical records	New York-power outage	↑ incidence in elderly, heat-related, respiratory	Lane et al. 2013 [95]; Anderson & Bell 2012 [105]
Water-borne disease	Household survey	Bangladesh-typhoon, Taiwan-typhoon	↑ incidence, hospitalizations in elderly, amoebiasis	Beier et al. 2015 [106], Lin et al. 2015 [107]
**Mental Health**				
Health-related quality of Life	SF-36 Health Survey (mental and physical components)	Gulf Coast USA hurricane-related flooding events; Sichuan, China	↓ in SF-36 scores	Cherry et al. 2017 [108]; Wu et al. 2015 [109]
Cognitive Impairment	Standardized testing	Japan-tsunami	↑ incidence for those in temporary housing, alleviated somewhat by walking and increased out of home activities	Ishiki et al. 2016 [110]
Post-traumatic stress disorder (PTSD)	Survey instruments	New Orleans-Hurricane Katrina, China	PTSD most prevalent psychiatric disorder; social support reduces symptoms	Dai et al. 2016 [111]; DeSalvo et al. 2007 [112]; Bei et al. 2013 [113]
